# Topical Treatment of Truncal Acne with Tretinoin Lotion 0.05% and Azelaic Acid Foam

**DOI:** 10.1155/2020/5217567

**Published:** 2020-03-16

**Authors:** Sharleen St. Surin-Lord, Judi Miller

**Affiliations:** ^1^Howard University College of Medicine, 520 W St NW, Washington, DC 20059, USA; ^2^Visage Clinical Research LLC, 1400 Mercantile Lane, Suite 110, Largo, MD 20774, USA; ^3^Strategic Pharmaceutical Advisors, 1750 Tysons Boulevard, Suite 1500 McLean, VA, USA

## Abstract

Truncal acne is present in approximately half of all patients with facial acne but is also occasionally seen in isolation. Important considerations when selecting treatment options for adult female acne, whether on the face, back, chest, or shoulders, include patient compliance, treatment response time, tolerability of the treatment, and psychosocial impact of the disease. Oral antibiotics are widely prescribed for truncal acne due to the challenges of applying topical therapy to such an extensive body surface area. In cases of severe inflammatory and nodular acne vulgaris, this may be a reasonable consideration; however, oral antibiotics should only be used for short durations. Overprescription contributes to microbial resistance and may cause disruption of the gastrointestinal microbiome. In many cases of mild, moderate, or even severe truncal acne, combinations of topical therapies may be valid alternatives. The introduction of foam formulations with enhanced percutaneous absorption and tretinoin lotion formulations that incorporate moisturizing/hydrating agents challenges the previously held idea that effective and tolerable treatment of truncal acne requires oral treatment. This case series describes four female African-American patients with truncal acne successfully treated with a combination of tretinoin lotion 0.05% and azelaic acid 15% foam.

## 1. Introduction

Acne vulgaris (AV) is characterized by lesions resulting from inflammation of pilosebaceous units [1]. Areas with the highest density of these units include the face, chest, and back. Cutibacterium acnes (C. acnes) is the major occupant of the pilosebaceous unit, accounting for up to 90% of the microbiota [2], and its overproliferation was previously thought to contribute to inflammatory acne. However, a rapidly expanding body of research indicates both the presence of a gut-brain-skin axis and that indigenous microbes of the skin and gut may be vital to the immunological, hormonal, and metabolic equilibrium of the host [3]. Thus, an efficacious and tolerable topical alternative to oral antibiotics in the treatment of AV warrants some investigation.

Truncal acne refers to AV affecting the chest and/or back and is present in approximately 50% of patients with facial acne [4]. In patients with acne, approximately 3% suffer only from truncal acne [5]. Pathophysiologically, truncal acne is no different from acne on the face, although the effects of pressure, occlusion, friction, and heat produced by clothing, shoulder pads, and other sporting equipment may contribute to exacerbation of acne on the chest, back, and shoulders [6]. Truncal acne can have a significant impact on sexual and bodily self-consciousness of appearance in both men and women [7].

Although truncal acne is expected to respond to therapy in a similar manner as acne on the face, the challenges of topical application, particularly to the back, are obvious, making patient compliance a challenge. As such, dermatologists have frequently resorted to prescribing oral antibiotics for these large and hard-to-reach body areas in contravention of the American Academy of Dermatology guidelines [8] and potentially increasing the growing problem of antibiotic resistance. It is currently estimated that more than 50% of C. acnes strains are resistant to topical macrolide antibiotics [9]; therefore, physicians have a duty to evaluate and prescribe appropriate alternative treatments.

## 2. Case Series

Patient 1 is a 30-year-old female who presented with a three-year history of severe back acne, frequent breakouts, and concerns regarding the dark spots they leave behind. She had seen a dermatologist in the past and had been treated with oral erythromycin, which improved but did not stop her breakouts. Switching to oral doxycycline did not help at all, nor did the use of adjuvant daily adapalene lotion 0.1%.

**Figure 1:**
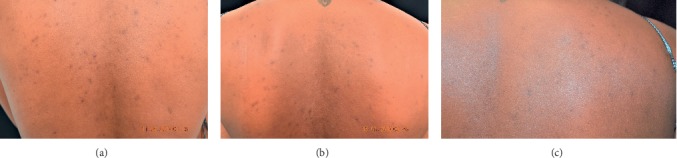
Patient 1: (a) pretreatment; (b) after 4 weeks treatment with tretinoin and azelaic acid; and (c) after 12 weeks treatment with tretinoin lotion and azelaic acid.

At baseline, the patient had no facial acne or hyperpigmentation, and the skin was smooth, soft, and radiant. AV was predominant on her chest and her back. Hyperpigmented macules were noted on her chest and back, along with hyperpigmented papules and pustules on her back (Figure 1(a)).

Since this patient had previously failed therapy with two oral antibiotics and adapalene, a retinoid of higher potency was required. Tretinoin lotion 0.05% (Altreno®, Ortho Dermatologics) was prescribed for its tretinoin concentration (0.05%), particle size (85% less than 10 microns), and homogenous distribution as well as its unique formulation with added hyaluronic acid and collagen, which we hoped would decrease dryness and irritation.

She was also prescribed azelaic acid foam 15% for twice daily use on her back in an effort to treat hyperpigmentation. Azelaic acid foam is indicated for rosacea and is known to have a lightening effect on the skin. It is an alternative for patients who wish to avoid hydroquinone.

The patient was followed up at 4 weeks. At this time, she verbalized satisfaction with the treatment. She stated that the dark spots looked much lighter and that she had not had an eruption since the initial visit. On physical exam, the postinflammatory hyperpigmentation was noticeably lightened. There was one pustule, one skin-colored papule, and one hyperpigmented papule. The patient denied any itching or burning sensations where the tretinoin 0.05% lotion had been applied, and there was no evidence of retinoid dermatitis or xerosis in the treatment areas. (Figure 1(b)).

At 12-week follow-up, there was no evidence of acne and sustained improvement of postinflammatory hyperpigmentation (Figure 1(c).

Patient 2 is a 33-year-old female with a history of acne for several years prior to her initial visit, as well as seborrheic dermatitis and mild atopic dermatitis. She had been on numerous over-the-counter (OTC) mediations and prescription medications for acne in the past. Initially, clindamycin phosphate and benzoyl peroxide gel 1.2%/2.5% (Acanya®, Coria Laboratories) was prescribed for her facial acne along with doxycycline 150 mg oral daily and azelaic acid foam 15% twice daily for her truncal acne. At 3-month follow-up, she complained that although the initial regimen helped, she was still experiencing eruptions on both her face and back. She was then treated with a benzefoam 9.8% topical foaming short contact cleanser for her face and chest, azelaic acid 15% foam for her chest, clindamycin phosphate and benzoyl peroxide gel 1.2%/3.75% (Onexton®, Ortho Dermatologics) for her face daily, and adapalene and benzoyl peroxide 0.3%/2.5% (Epiduo®, Galderma) topical gel nightly. She was prescribed hydroquinone 4% lotion twice daily for the hyperpigmentation on her chest and back. Three months later, she stated that the oral antibiotics were no help at all, and they were discontinued. Adapalene 0.1% lotion was prescribed for application to the chest and back once daily.

This regimen successfully controlled the facial and truncal acne for over 6 months, until the truncal acne recurred. At this time, she was commenced on minocycline 115 mg orally daily, but as this did not control the truncal acne well enough, the patient utilized a recommended 10% glycolic acid cleanser to her chest and back three times per week and a 2% glycolic and 2% salicylic acid solution to spray on her trunk along with the adapalene 0.1% lotion.

The hyperpigmentation on the patient's chest, shoulders, and back remained quite remarkable, and the patient became frustrated with the dyspigmentation. The oral antibiotic was discontinued, and a series of 20% salicylic acid chemical peels were performed monthly for a total of 5. These were effective in lightening the hyperpigmented macules and controlling the truncal acne at first; however, upon follow-up, truncal acne and dark spots prevailed over facial acne. Therapy with tretinoin lotion 0.05% was initiated twice daily, along with azelaic acid 15% foam. The patient was asked to return in 4 and then 8 weeks. Truncal acne was well controlled, as was postinflammatory hyperpigmentation. The patient denied any itching or burning sensations where the tretinoin lotion was applied, and there was no evidence of retinoid dermatitis or xerosis in the treatment areas.

**Figure 2:**
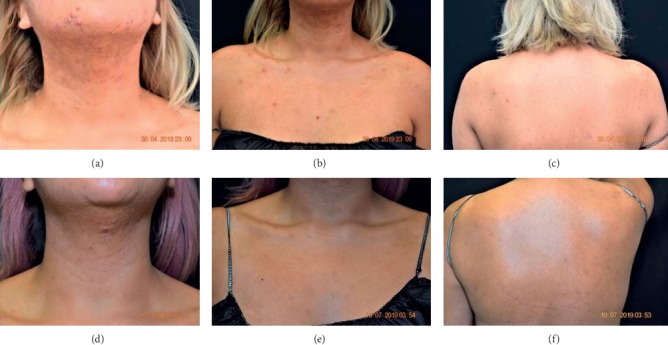
Patient 3: (a) pretreatment (chin and neck); (b) pretreatment (chest); (c) pretreatment (back); (d) after 12 weeks treatment with tretinoin lotion and azelaic acid (chin and neck); (e) after 12 weeks treatment with tretinoin lotion and azelaic acid (chest); and (f) after 12 weeks treatment with tretinoin lotion and azelaic acid (back).

Patient 3 is a 24-year-old female who presented with a history of moderate facial acne since age 13. She stated that since starting oral contraceptives two months before, her skin had become sensitive to touch and that acne bumps had become bigger. A change in diet helped mildly but breakouts remained consistent. Two weeks prior, she had a severe flare with lumps all over her neck, jawline, and chest. Her cellphone camera photos were reviewed which revealed numerous inflamed papules on the face and nodules on the neck. On physical examination, there were erythematous papules on the nuchal scalp and thick flakes with some erythema. There were inflamed papules, some atrophic skin, and pustules on the cheeks, with significant pustules and inflamed papules on her chin. There was some scarring, acne cysts, and erythematous macules on the neck, with many hyperpigmented macules. The patient's chest had erythematous papules and macules, while her back and shoulders had many erythematous papules and hyperpigmented macules (Figures 2(a)–2(c)).

The patient declined oral isotretinoin at this time. Due to the inflammatory nature of this patient's presentation, she was initially prescribed tazarotene 0.1% (Fabior®, Mayne Pharma) foam nightly with clindamycin phosphate and benzoyl peroxide gel 1.2%/3.75% in the morning for her facial acne. She was instructed to use Neutrogena Hydro Boost to treat any dry skin or flaking. For the truncal acne, she was instructed to apply tretinoin 0.05% lotion nightly to the chest, neck, shoulders, and back and azelaic acid 15% foam twice daily to the chest, shoulders, and back. She did not wish to treat the seborrheic dermatitis. She was also prescribed sarecycline 100 mg by mouth daily. The patient presented 7 days later complaining of a generalized pruritic eruption. On physical examination, there were still inflamed papules and cysts on the patient's facial skin, and her chest, back, arms, and legs had developed a morbilliform eruption. The sarecycline was discontinued, and the patient was placed on a short course of oral corticosteroids as well as fluocinonide 0.05% topical cream for twice daily use and instructed to never take oral tetracyclines again. 3 weeks later, the patient stated that her skin was clear from drug eruption. The Neutrogena Hydro Boost that was recommended to use in case of dryness caused a burning sensation on her face after using azelaic acid foam at night, so she changed foam frequency to every other night but continued using the foam twice daily and tretinoin lotion 0.05% every night on her chest and back. Upon physical exam, the following were noted: scales on the scalp and some cysts, inflamed papules, and hyperpigmented macules on the face but less than before. On the chest, shoulders, and back, there were fewer acne papules than at the previous visit. The patient stated that she was satisfied with her current regimen; however, glycolic acid 10% wipes were recommended for morning application to better control facial acne. This patient was seen one month later, at which time her face, chest, and neck were clear. (Figure 2(d))

Patient 4 is a 32-year-old female who has been treated for moderate facial acne for the past three years with a regimen that included tazarotene 0.1% gel, dapsone 7.5% gel, and clindamycin phosphate and benzoyl peroxide gel 1.2%/3.75%. Despite this aggressive topical regimen, this patient never completely cleared. In 2017, she sought treatment for her truncal acne as she began to notice eruptions on her chest and back after she began to work out. Physical examination revealed erythematous papules and inflamed papules on her cheeks, forehead, and chin, as well as skin-colored and hyperpigmented papules on her chest, back, and shoulders. She was advised to shower immediately after workouts, and the following were added to her treatment regimen: benzefoam 9.8% topical to the chest and back and azelaic acid foam 15% daily to the face. She continued to flare over the next three months with erythematous papules and inflamed papules on her cheek, forehead, and chin and notably with skin-colored and hyperpigmented papules on her chest, back, and shoulders. She declined any oral medications, so it was advised that she uses Cetaphil cleansing cloths immediately after working out to cleanse her skin and then shower with the benzefoam short contact cleanser soon after. Subsequently, she agreed to try minocycline 115 mg orally daily and adapalene 0.1% lotion on her back once daily. At 2-month follow-up, the flares were under control and the patient only had hyperpigmentation on her back, shoulders, and chest. Minocycline was changed to doxycycline, but 4 months later, truncal acne began to flare again. The patient was offered, but declined, isotretinoin therapy. Over the next 12 months, the patient continued to flare on her face and trunk and also developed atopic dermatitis. She eventually admitted to not being adherent to oral regimens and stated she preferred topical regimens, so tretinoin lotion 0.05% once daily at bedtime and azelaic acid 15% foam twice daily were initiated for her truncal acne. At one-, two-, and 3-month follow-ups, she expressed satisfaction with not having to use oral medications and stated she had broken out less between visits.

## 3. Discussion

Although AV is principally a disorder of adolescence, research suggests that the prevalence of adult acne is increasing, especially in females [10]. In this population, persistent acne, i.e., acne that continues from adolescence into adulthood is seen in about 80% of cases. Important considerations when selecting a treatment for adult female acne, whether on the face or trunk, are the slower response to treatment, increased likelihood of skin irritation, and greater psychosocial impact of the disease [11]. Oral antibiotics, although widely prescribed for the back and truncal acne, should be used only when absolutely necessary and for short durations. Not only does overprescription contribute to microbial resistance, antibiotics can disrupt gut flora and the gut-skin axis and the resulting intestinal dysbiosis, and worsening of acne [12].

Topical retinoids are highly effective and are recommended as first line therapy in mild-to-moderate acne; however, they can be associated with significant skin dryness, erythema, and even development of dermatitis [13]. As such, successful treatment must always balance efficacy against tolerability, and less irritating formulations may provide the best results. In the author's experience, many patients have not tolerated higher-dose retinoids on the chest and back, making adapalene 0.1% lotion a good option. However, when this is not effective, one must consider other, more potent, and tolerable treatment options. The most recent of these is a topical lotion formulation of tretinoin 0.05% together with sodium hyaluronate, soluble collagen, glycerin, and small amounts of mineral oil. These moisturizing/hydrating agents have been shown to enhance efficacy, alleviate dryness and improve skin comfort, and accelerate the healing process [14].

While previous studies have shown that azelaic acid therapy can enhance efficacy and improve patient satisfaction when used in combination with other topical medications such as benzoyl peroxide 4% gel, clindamycin 1% gel, tretinoin 0.025% cream, and erythromycin 3%/benzoyl peroxide 5% gel [15], this is the first report documenting the use of a combination of once daily application of topical tretinoin 0.05% lotion and twice daily azelaic acid 15% foam in truncal acne. In addition to the treatments noted, all patients were instructed to use a sunscreen with SPF 50 on the chest and back if those areas were ever exposed and daily on the face, except patient 1 who only had truncal acne.

It should be noted that none of these patients were taking hormone based therapies, i.e., combined oral contraceptives and/or spironolactone as part of their adult acne treatment regimen, although Patient 3 was taking a combined norgestimate and ethinyl estradiol pill as an oral contraceptive. We acknowledge that while hormonal therapies are usually highly effective for patients with severe seborrhea, flare-ups before menstruation, endocrine abnormalities, and persistent inflammation recalcitrant to treatment, they are not without side effects and spironolactone is not approved for use in acne in the US.

In the four female adult patients reported here, this combination of tretinoin 0.05% lotion and azelaic acid 15% foam resulted in sustained improvement of their long-standing, previously treatment refractory truncal acne during the follow-up observation period. Nevertheless, we acknowledge that these results may be limited by the relatively short follow-up, especially as adult female acne tends to be more chronic and relapse more frequently than adolescent acne, and that prolonged maintenance treatment will likely be essential. Additionally, these results need to be confirmed in a larger patient population.
